# c-Src and Neural Wiskott-Aldrich Syndrome Protein (N-WASP) Promote Low Oxygen-Induced Accelerated Brain Invasion by Gliomas

**DOI:** 10.1371/journal.pone.0075436

**Published:** 2013-09-19

**Authors:** Zhuo Tang, Lita M. Araysi, Hassan M Fathallah-Shaykh

**Affiliations:** 1 Department of Neurology, The University of Alabama at Birmingham, Birmingham, Alabama, United States of America; 2 Department of Mathematics, The University of Alabama at Birmingham, Birmingham, Alabama, United States of America; 3 Department of Cell, Developmental and Integrative Biology, The University of Alabama at Birmingham, Birmingham, Alabama, United States of America; 4 Department of Biomedical Engineering, The University of Alabama at Birmingham, Birmingham, Alabama, United States of America; 5 Department of Mechanical Engineering, The University of Alabama at Birmingham, Birmingham, Alabama, United States of America; 6 The UAB Comprehensive Neuroscience Center, Birmingham, Alabama, United States of America; 7 The UAB Comprehensive Cancer Center, Birmingham, Alabama, United States of America; University of Florida, United States of America

## Abstract

Malignant gliomas remain associated with poor prognosis and high morbidity because of their ability to invade the brain; furthermore, human gliomas exhibit a phenotype of accelerated brain invasion in response to anti-angiogenic drugs. Here, we study 8 human glioblastoma cell lines; U251, U87, D54 and LN229 show accelerated motility in low ambient oxygen. Src inhibition by Dasatinib abrogates this phenotype. Molecular discovery and validation studies evaluate 46 molecules related to motility or the src pathway in U251 cells. Demanding that the molecular changes induced by low ambient oxygen are reversed by Dasatinib in U251 cells, identifies neural Wiskott-Aldrich syndrome protein (NWASP), Focal adhesion Kinase (FAK), 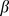-Catenin, and Cofilin. However, only Src-mediated NWASP phosphorylation distinguishes the four cell lines that exhibit enhanced motility in low ambient oxygen. Downregulating c-Src or NWASP by RNA interference abrogates the low-oxygen-induced enhancement in motility by *in vitro* assays and in organotypic brain slice cultures. The findings support the idea that c-Src and NWASP play key roles in mediating the molecular pathogenesis of low oxygen-induced accelerated brain invasion by gliomas.

## Introduction

Motility is not only critically relevant to the understanding and therapeutics of cancer but is also important in several pathological processes including vascular disease, osteoporosis, rheumatoid arthritis, and mental retardation. Tumor cell migration and invasion involves highly coordinated steps of dissociation of existing cellular adhesions, remodeling the actin cytoskeleton to project lamellipodium extensions, formation of new adhesions, and tail detachment along with proteolytic processing and secretion of extracellular matrix proteins along the trajectory [1].

Malignant gliomas are notorious not only because of their resistance to conventional chemotherapy and radiation therapy but also for their ability to invade the surrounding brain, thus causing neurological impairment and significant morbidity from cognitive deficits and limitations of mobility. Brain invasion, a hallmark of gliomas, also helps glioma cells evade therapeutic strategies. In particular, the recent use of Bevacizumab, an antiangiogenic drug, for the treatment of gliomas has led to new insights on tumor recurrence by brain invasion and to the development of the RANO criteria (Response Assessment in Neuro-Oncology working group)[2], [3], [4], [5], [6]. There is current interest in the idea that glioma cells, sensing a hypoxic environment, react by aggressive migration and brain invasion; this ability is called the ‘grow-or-go’ phenotype. Keunen et al. studied glioblastoma (GBM) xenografts in animal brains and showed that treatment with Bevacizumab lowered blood supply but was associated with an increase in infiltrating tumor cells [7]. Here, we use the term *phenotype* to mean low oxygen-induced enhancement in motility.

Hypoxia is a term used to describe reduced levels of oxygen and can be defined as a condition in which the oxygen pressure in the environment is less than 5 to 10 mmHg [8]. Hypoxia typically ranges from 0.1 percent to 3 percent oxygen, with exact definitions varying according to individual researchers [9], [10], [11], [12]. Normoxia for tissue culture experiments is considered approximately 21 percent oxygen. In more general terms, tissue hypoxia occurs whenever there is an inadequate supply of oxygen to meet consumption. Although indirect evidence for hypoxia in human tumors was first reported in the 1950s, Peter Vaupel and colleagues were among the first researchers to demonstrate direct evidence of hypoxia in human cancers, as well as linking hypoxia with increased metastasis and poor prognosis in patients with squamous tumors of the head and neck, cervical cancers, and breast cancers [13], [14], [15],[16].

Hypoxia-inducible factor (HIF) is a transcription factor that plays a central role in mediating the ability to adapt to low-oxygen concentrations [9], [10]. One of the primary cellular events in response to the initial exposure to hypoxia is activation of hypoxia-inducible factor 1 (HIF-1), a hetero-dimeric basic helix-loop-helix protein, composed of 2 subunits: HIF-1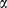, which is up-regulated in an oxygen-dependent manner, and HIF-1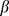, which is constitutively expressed [17], [18], [19]. Over-expression of HIF-1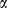 is seen in many cancer types associated with a poor prognosis, like malignancies of the brain, oropharynx, breast, cervix, ovary, and uterus [20], [21]. Since we observe a HIF-1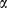 response in glioma cells at 5% oxygen (see below), we evaluate the phenotype of low-oxygen mediated hypermotility at both 5% and 1%, because enhanced motility at 5% ambient oxygen implies an increased propensity toward invasion.

The molecular pathogenesis of low oxygen-induced hypermotility remains unknown. Genome-scale expression discovery by microarrays identified a putative large network that appears to be related to glioma motility [22]. Here, we show that 4 of 8 glioma cell lines exhibit enhanced motility in low oxygen conditions. Furthermore, by evaluating the elements of this network by protein assays, RNA interference, and motility assays including time-lapse microscopy in live brain sections, we obtain evidence that identifies c-Src and neural Wiskott-Aldrich syndrome protein (NWASP) as key mediators.

The presentation is organized as follows; we begin by showing that four of eight GBM cells lines exhibit enhanced motility in 5% ambient oxygen. This phenotype is also observed in 1% ambient oxygen. Interestingly, Dasatinib, a Src inhibitor, abrogates the low oxygen-induced hypermotility in the four cell lines that exhibit this phenotype. In addition, downregulating c-Src by RNA interference (siRNA) abrogates this phenotype. To discover molecules that promote this phenotype, we study 46 proteins related to the c-Src network in the presence of either Dasatinib or vehicle and with or without hypoxia; neural Wiskott-Aldrich syndrome protein (NWASP), Focal adhesion Kinase (FAK), 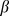-Catenin, and Cofilin are discovered by imposing logical rules. Findings from experiments, which downregulate src expression by siRNA, identify NWASP as the only molecule that distinguishes the cell lines that exhibit enhanced motility in hypoxic conditions. Finally, we show that downregulating NWASP expression by siRNA abrogates this phenotype in 5% as well as 1% ambient oxygen.

## Materials and Methods

### 3.1 Cell culture

Human glioma tumor cell lines U251 (see [23]), U87 (see [23]), U373 (see [23]), D54 (see [24]), LN229 (see [25]), LN319 (see [26]), LN308 (see [26]), and SNB19 (see [27]) are a generous gift from Yancey Gillespie, Ph.D, The University of Alabama at Birmingham. Cultures were confirmed to be mycoplasma free by PCR.

### 3.2 Animal handling

Newborn athymic nude mice were purchased from Harlan Laboratories and housed at University of Alabama at Birmingham under specific pathogen-free conditions. Animal experiments were carried out after approval and in accordance with guidelines from the University of Alabama at Birmingham Animal Resources Program.

### 3.3 Hypoxia chamber

Cultured cells were incubated in a Biospherix hypoxia chamber with an oxygen-controlled glove box (Lacona, NY).

### 3.4 siRNA transfection

Transfection experiments were done in triplicates; 10 uM of Silencer select SRC siRNA (Ambion, Austin, TX, USA) targeting human v-Src mRNA (sense GCCUCUCAGUGUCUGACUUtt, antisense AAGUCAGACACUGAGAGGCag) and two human NWASP siRNAs (sense CGACAGGGUAUCCAACUAAtt, antisense UUAGUUGGAUACCCUGUCGta) and (sense GGAAUUGUGGGUGCAUUAAtt, antisense UUAAUGCACCCACAAUUCCtg) were transiently transfected using SE Cell Line 4D-Nucleofector X Kit L, program CM-137 (AMAXA, Koeln, Germany), following the manufacturer's instructions. A control experiment was performed in parallel using silencer negative control siRNA (Ambion, Austin, TX). Transfection efficiency was routinely 85 to 90%, as determined by fluorescent microscopy. For immunoblotting, cell lysates were prepared at 72 hours after siRNA transfection.

### 3.5 Immunoblotting

Notice that the protein expression/phosphorylation assays are done in triplicates because protein measurements are noisy (*ie* may be false positive). Cells were lysed in immunoprecipitation assay buffer (Boston BioProducts, Ashland, MA, USA) with protease inhibitor cocktail, EDTA-Free (Thermo Scientific, Rockford, IL, USA), and phosphatase inhibitor cocktail (Calbiochem, Darmstadt, Germany). Protein concentrations were determined by the BCA protein assay kit (Thermo Scientific, Rockford, IL, USA). Samples were separated by sodium dodecylsulfate-polyacrylamide gel electrophoresis on a 10% polyacrylamide gel and transferred onto nitrocellulose membrane (Thermo Scientific, Rockford, IL, USA) using a transfer tank. Immunodetection was performed using the following primary antibodies from Cell Signaling (Danvers, MA, USA): AKT, p-AKT (S473), 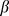-Catenin, Cofilin, p-Cofilin (Ser3), Cortactin, p-Cortactin (Tyr421), EGFR, p-EGFR (Tyr845), p-EGFR (Tyr992), p-EGFR (Tyr1068), FAK, p-FAK (Tyr397), p-FAK (Tyr576/577), HIF-1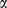, p44/42MAPK, p-p44/42 MAPK(Thr202/Tyr204), MyPT1, p-MYPT1(Thr853), Notch1, Cleaved Notch1, PAK1/2/3, p-PAK1(Thr423)/PAK2(Thr402), P53, PP5, Src, p-Src(Tyr416), p-Src (Tyr527), Stat3, p-Stat3(Tyr705), and GAPDH. The following antibodies were obtained from Abcam (Cambridge, MA, USA): ASAP1/DDEF1, Dynamin1, Dynamin2, E Cadherin, p-E Cadherin (Ser838+Ser840), Hes1, IGF1R, p-IGF1R (Tyr1158), p-IGF1R(Tyr1161), Nck1/2, NWASP, p-N WASP (Tyr256), RBPJK, WIPF1. Antibodies for DEC1 and DEC2 and HIF2 were obtained from Santa Cruz Biotechnology (Santa Cruz, CA, USA); actin antibody was purchased from (Sigma-Aldrich, St. Louis, MO, USA). The reaction was developed by western lightning plus ECL enhanced chemiluminescence substrate (PerkinElmer, Waltham, MA, USA). The signals were quantified by the NIH ImageJ (http://rsbweb.nih.gov/ij/).

### 3.6 *In vitro* invasion assays

The invasiveness of glioma cell lines was assayed by two *in vitro* assays, the Millipore QCM Collagen-Based cell invasion and the BD BioCoat Matrigel invasion chamber assay. Briefly, the collagen-based invasion assay is based on the Boyden Chamber principle; cell suspensions (1.25×10^4 ^cells/mL serum-free medium) were added to the top chamber, and complete medium was added to the lower chamber. For controls, both the top chamber and the lower chamber contained serum-free medium. The noninvading cells on the upper surface of membrane were removed from the chamber by gentle scrubbing with a cotton swab, and the invading cells on the bottom of the insert membrane were incubated with the Cell Stain Solution, extracted, then detected on a TECAN infinite microplate reader M200 (TECAN, Männedorf, Switzerland) at 560 nm. For the second assay, 24-well BioCoat matrigel invasion chambers (Becton-Dickinson, Bedford, Massachusetts, USA) were rehydrated following to the manufacturer's instructions. Cell suspensions (1.25×10^4 ^cells/mL serum-free medium) were added to the top chamber, and complete medium was added to the lower chamber. For controls, inserts without matrigel were used. The noninvading cells on the upper surface of membrane were removed from the chamber by gentle scrubbing with a cotton swab, and the invading cells on the lower surface of the membrane were stained with the Diff-Quick stain kit (Becton-Dickinson). After 2 washings with water, the chambers were allowed to air dry and membranes were mounted on glass slides and 5 high power fields were counted manually (40x magnification). The number of invading cells was expressed as the mean number of the cells invading through the Matrigel insert membrane divided by the mean number of cells migrating under the control insert membrane conditions multiplied by 100. Cells were incubated at 37°C in 5% carbon dioxide and 1%, 5% or 21% oxygen for 48 hours. All assays were performed in triplicates.

### 3.7 Organotypic brain slice cultures

For detailed information on the protocol for organotypic brain slice cultures, see [28], [29], [30]; newborn pups are used because adult brains do not yield live sections. Briefly, the brains of P7 nude mouse pups were injected by 3×10^5^ cells in 3 *µ*l (see [31]), using a Hamilton syringe and a needle, inserted 3.5 mm (1 mm anterior and 2 mm to the right of the bregma) into the brain. The brains were harvested two days after intracerebral tumor injection and quickly immersed in ice-cold Hank's balanced salt solution (Invitrogen), supplemented with glucose (41.55 mM), antibiotic and antimycotic agents (1 g/mL penicillin, 1 g/mL streptomycin, 0.25 g/mL amphotericin B). The brain was dissected, and 200 µm-thick transverse slices were prepared using a custom-designed tissue slicer strung with 20 µm-thick tungsten wire (California Fine Wire). After 30 min incubation at 4°C, slices were plated on tissue culture plate inserts (Millicell-CM, Millipore), fed with culture medium, and placed immediately in an incubator at 37°C, 5% CO2, and 95% relative humidity. The culture medium consisted of 50% Minimum Essential Medium, 25% Hank's balanced salt solution, 20% heat-inactivated equine serum, 1 mM L-glutamine, and 36 mM D-glucose.

### 3.8 Time-lapse microscopy

Tumor cell motility in live sections were performed using the Nikon TI PERFECT FOCUS inverted imaging system with XY and Z automation (motorized stage) and fluorescence, fitted with a live cell chamber with temperature, humidity, and gas mixing control (okolabs, www.oko-lab.com), the CoolSNAP HQ2 Monochrome Camera, and acquisition and analysis software. The set-up permits temperature, oxygen, and *CO_2_* control in the tissue culture chamber that fits on the microscope stage.

### 3.9 Statistical analysis

p-values are computed by a two-sample two-tailed Student's T-test (ttest).

## Results

### 4.1 Low oxygen-induced hypermotility in glioma cell lines and effects of src inhibition

**Figure 1:**
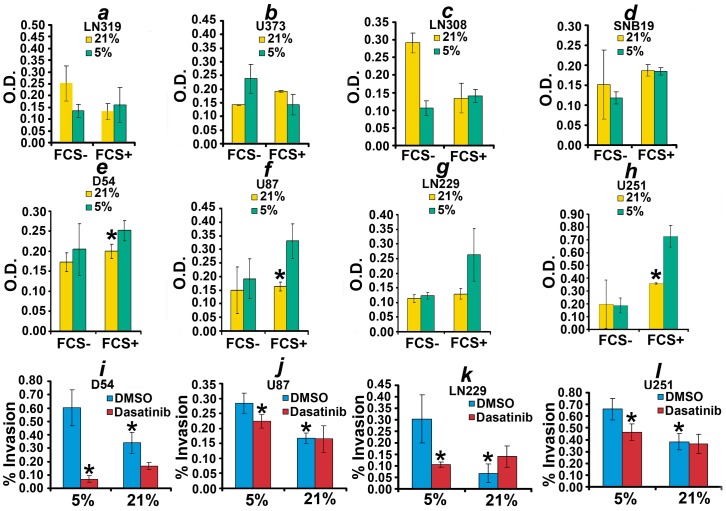
Low oxygen enhances motility in four of eight GBM cell lines. (a)-(h) plot the motility of the 8 human GBM cell lines, assayed by the colorimetric in vitro assay. Four cell lines, U251, U87, LN229, and D54, exhibit enhanced motility in 5% oxygen as compared to normoxia. FCS− and FCS+ indicate the absence and presence of fetal calf serum in the lower chamber, respectively. FCS serves as a chemoattractant that gives a direction of movement. The absence of fetal calf serum in the lower chamber serves a negative control. * in (e), (f), and (h) indicate a two-sample two-tailed ttest *p*<0.05 as compared to 5% oxygen in the presence of FCS. The two-sample two-tailed ttest p-value for (g) is 0.06. D54, U87, LN229, and U87 were also evaluated by a second in vitro motility assay in 5% or 21% ambient oxygen in the presence of Dasatinib or vehicle (i)-(l). Cells were cultured in the presence of Dasatinib at 100 nM or vehicle (DMSO). * in (i)-(l) indicate a two-sample two-tailed ttest *p*<0.05 as compared to 5% DMSO.

We study the motility of eight human GBM cell lines in 21% and 5% ambient oxygen by a colorimetric assay *in vitro* assay (Figures 1a-h); the results reveal that U251, U87, LN228, and D54 exhibit enhanced invasion in 5% as compared to 21% oxygen. This enhancement in invasion is statistically significant for U251, U87, and D54 (*p*<0.05) and the p-value for LN229 is 0.06. To enhance our confidence that the four cell lines exhibit enhanced invasion in low ambient oxygen, we evaluate motility by the matrigel invasion assay. The results confirm the findings of the first assay; in particular, U251, U87, LN228, and D54 cells show a statistically significant enhancement in invasion in 5% as compared to 21% ambient oxygen (Figures 1i-l). Notice that the presence of fetal calf serum in the lower chamber serves as a chemoattractant that gives a direction of movement.

Microarray expression discovery suggested that Src may play a role in glioma motility [22]. We, therefore, evaluate the effects of Dasatinib, a Src kinase inhibitor, on this phenotype. The findings reveal that Dasatinib abrogates the augmentation of motility of U251, U87, LN228, and D54 observed in 5% ambient oxygen conditions (see Figures 1i-l).

**Figure 2:**
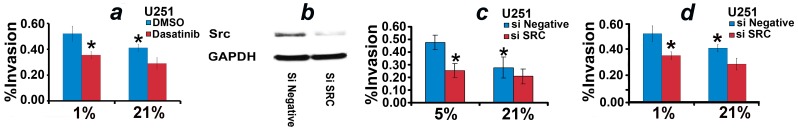
Effects of Src siRNA and 1% oxygen on motility of U251 cells. (a) plots the matrigel invasion assay of U251 cells in 1% and 21% ambient oxygen in the presence of Dasatinib (100 nM) or vehicle (DMSO). * indicates a two-sample two-tailed ttest *p*<0.05 as compared to 1% DMSO. (b) displays western blots showing downregulation of c-src by siRNA. (c) plots the matrigel invasion data of U251 cells transfected by negative control (blue) and c-src siRNA (red) in 5% and 21% ambient oxygen. (d) plots the matrigel invasion data of U251 cells transfected by negative control (blue) and c-src siRNA (red) in 1% and 21% ambient oxygen. * indicates a two-sample two-tailed ttest *p*<0.05 as compared to 1% or 5% negative control siRNA.

Interestingly, U251 cells also exhibit enhanced motility in 1% ambient oxygen as compared to 21% and this enhancement in motility is also abrogated by Dasatinib (see Figure 2a). Next, we specifically target c-src expression by siRNA in U251 cells cultured in 21%, 5% or 1% ambient oxygen conditions. The results confirm that downregulating c-src abrogates the phenotype of enhanced motility induced by both 5% and 1% oxygen levels (see Figure 2b-d). We conclude that Src appears to be a key mediator of low oxygen-induced enhancement of glioma motility.

### 4.2 Molecular discovery/validation

**Figure 3:**
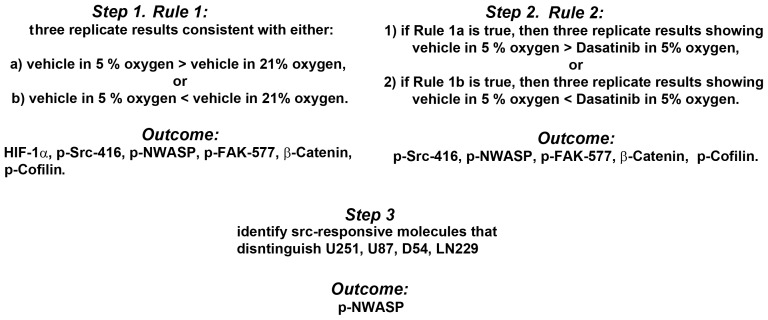
Discovery steps and outcomes. The goal of step 1 is to isolate the molecules that are regulated by 5% ambient oxygen in U251 cells. The goal of step 2 is to identify the molecular events of step 1 in U251 cells, which that are abrogated by Dasatinib. The third step isolates the molecules that distinguish the four cell lines that show enhanced motility in 5% ambient oxygen.

**Figure 4:**
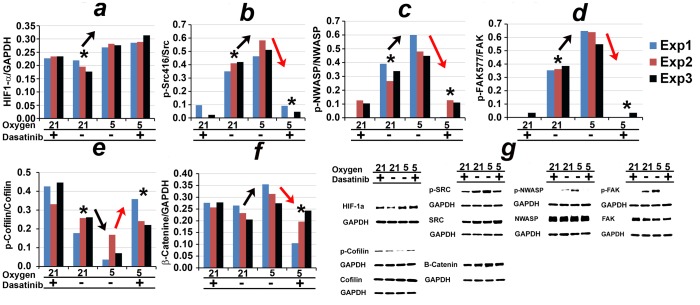
Molecular discovery in U251 cells. Shown are the expression levels of molecules, p-Src-416, p-NWASP, p-FAK-577, p-Cofilin, and 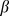-Catenin, which survived *Rules 1* and *2* (see Figure 3). Normalized protein expression ratios from each of the three experiments is indicated by a different color (Exp1, Exp2, Exp3). The y-axes of (a)-(f) indicate 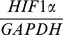, 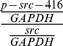, 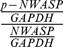, 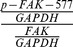, 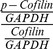, and 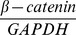, respectively. (g) shows representative western blots. * indicates a two-sample two-tailed ttest *p*<0.05 in the group indicated as compared to vehicle in 5%. Black and red arrows indicate Rules 1 and 2, respectively.

Because motility is a multistep process; it likely requires the activation of molecules downstream of Src. To discover these elements, we select 46 molecules, chosen predominantly from the motility network reported in [22] and from effectors proteins downstream of Src (see Table 1 for a list). Molecular discovery is done in a single cell line (U251, see Figures 3 and 4); nevertheless, once a target is identified, we validate its biological effects in the other cell lines. Because the phenotype of enhanced motility is induced by low ambient oxygen, we looked for molecular events that are caused by 5% ambient oxygen. We select 5% instead of 1%, because it is biologically significant as it enhances motility and upregulates HIF1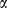 in U251 cells (see below) and since it indicates increased propensity towards invasion.

**Table 1:** A list of the molecules.

**AKT**	**Protein Kinase B 1, 2, and 3**
p-AKT	Phospho-AKT (S473)
ASAP1/DDEF1	Development and Differentiation-Enhancing Factor 1
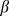 -Catenin	Cadherin-Associated Protein, Beta
Cofilin	Cofilin, CFL1
p-Cofilin	Phospho-Cofilin (Ser3)
Cortactin	Oncogene EMS1
p-Cortactin	Phospho-Cortactin (Tyr421)
DEC1	Basic Helix-Loop-Helix Family, Member E40; BHLHE40
DEC2	Basic Helix-Loop-Helix Family, Member E41; BHLHE41
Dyn1	Dynamin 1
Dyn2	Dynamin 2
E-Cadherin	Cadherin 1
p-E-Cadherin	Phospho-E-Cadherin (Ser838+Ser840)
EGFR	Epidermal Growth Factor Receptor
p-EGFR 845	Phospho-EGFR (Tyr845)
p-EGFR 992	Phospho-EGFR (Tyr992)
p-EGFR 1068	Phospho-EGFR (Tyr1068)
FAK	Focal Adhesion Kinase
p-FAK 397	Phospho-FAK (Tyr397)
p-FAK 577	Phospho-FAK (Tyr577)
Hes1	Hairy/Enhancer OF Split, Drosophila, Homolog of, 1
HIF-1 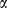	Hypoxia-Inducible Factor 1, Alpha Subunit
IGF1R	Insulin-Like Growth Factor 1 Receptor
p-IGF1R 1158	Phospho-IGF1R (Tyr1158)
p-IGF1R 1161	Phospho-IGF1R (Tyr1161)
MAPK	P42/p44 Mitogen-Activated Protein Kinase (Erk1/Erk2)
p-MAPK	Phospho- MAPK (Erk1/Erk2) (Thr202/Tyr204)
MYPT1	Myosin phosphatase Target subunit 1
p-MYPT1	phospho-MYPT1 (Thr853)
NCK1/2	Nck Adaptor Protein 1/2
Notch 1	Notch 1, Drosophila
Cleaved Notch 1	Cleaved Notch 1 (Val1744)
PAK	p21-Activated Kinases 1/2/3
p-PAK	Phspho-Pak1 (Thr423)/Pak2(Thr402)
p53	Tumor Protein p53
PP5	Protein phosphatase 5
RBPJK	Recombination Signal-Binding Protein For Immunoglobulin Kappa J Region
Src, V-Src	Avian Sarcoma (Schmidt-Ruppin A-2) Viral Oncogene
p-Src 416	Phospho-Src family (Tyr416)
p-Src 527	Phospho-Src family (Tyr527)
Stat3	Signal Transducer And Activator of Transcription 3
p-Stat3	Phospho-Stat3 (Tyr705)
NWASP	Wiskott-Aldrich Syndrome Gene-Like WASL
p-NWASP	Phospho-NWASP (Tyr256)
WIPF1	WAS/WASL-Interacting Protein Family, Member 1

The molecular discovery is done in three steps. First, we detect the molecules regulated by 5% ambient oxygen in U251 cells; the second step identifies the molecular events of step 1, which are abrogated by Dasatinib (see Figure 3). The third step isolates the molecules that distinguish the four cell lines that show enhanced motility in 5% ambient oxygen. The first rule demands that either: 1) all three replicate molecular data are higher in 5%, or 2) all three replicate data are lower in 5% ambient oxygen, as compared to normoxia (*rule 1*, Figure 3). For example, a protein is filtered (not considered) if one of the three replicates shows that its expression level is higher in 5%, while the remaining two show lower expression levels in 5% as compared to 21% ambient oxygen. Six of 46 molecules survived this rule (see Figures 3 and 4), including HIF1-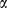, p-Src-416, p-NWASP, p-FAK-577, 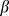-Catenin, and p-Cofilin. In particular, the level of p-Cofilin significantly decreases, while the remaining five molecules are significantly upregulated in 5% as compared to 21% ambient oxygen.

Because Dasatinib inhibits the phenotype of enhanced motility and invasion in low ambient oxygen (see Figure 1), we were interested in determining which of the six molecular changes, caused by low oxygen, are reversed by Dasatinib (see Figure 3). Therefore, we impose a second rule demanding that: 1) if the expression level of a protein increases (decreases) in low oxygen, then it should decrease (increase) in the presence of Dasatinib (see arrows in Figure 4). Five of the six molecules, except HIF-1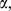 show a Dasatinib-mediated reversal of the low-oxygen induced molecular changes. Notice that in the cases of p-Src-416, p-NWASP, p-FAK-577, and 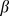-catenin, Dasatinib lowers the elevated expression levels (p<0.05); while, the expression level of p-Cofilin is increased in the presence of Dasatinib (p<0.05, Figure 4 black vs. red arrows). Observe also that the expression level of HIF-1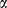 does not decrease in the presence of Dasatinib (Figure 4).

**Figure 5:**
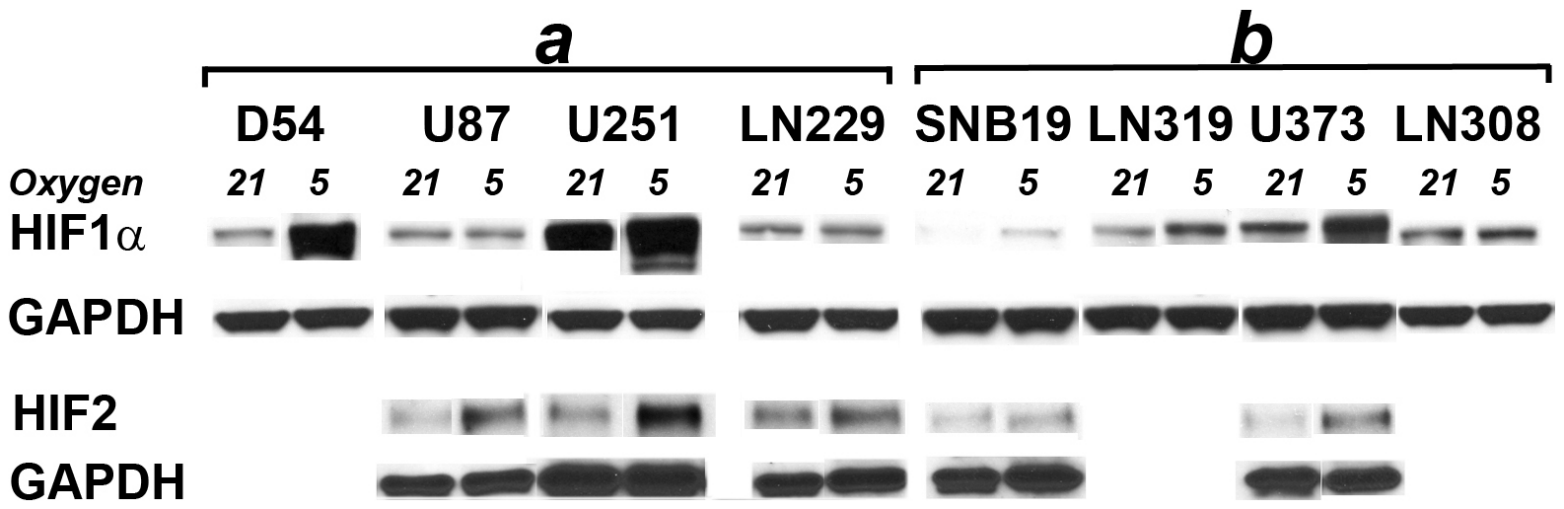
HIF1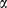 and HIF2 expression. Western analysis of HIF1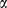 and HIF2. 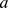 and *b* indicate the cell lines that do and do not exhibit the phenotype of increased motility under hypoxic conditions, respectively.

The discovery steps, detailed above, (see Figure 3) suggest that HIF1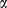 does not play a central role in mediating the low oxygen-mediated enhancement in motility. To confirm, we study the expression of HIF1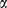 and HIF2 in cells lines that do and do not exhibit this phenotype in 21% and 5% ambient oxygen. As expected, the results reveal that HIF1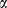 and HIF2 do not distinguish U251, U87, D54 and LN229 from the other cell lines. In particular, 5% oxygen upregulates: 1) HIF1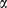 in SNB19 and U373, and 2) HIF2 in U87, U251, LN229 as well as SNB19 and U373 (see Figure 5).

**Figure 6:**
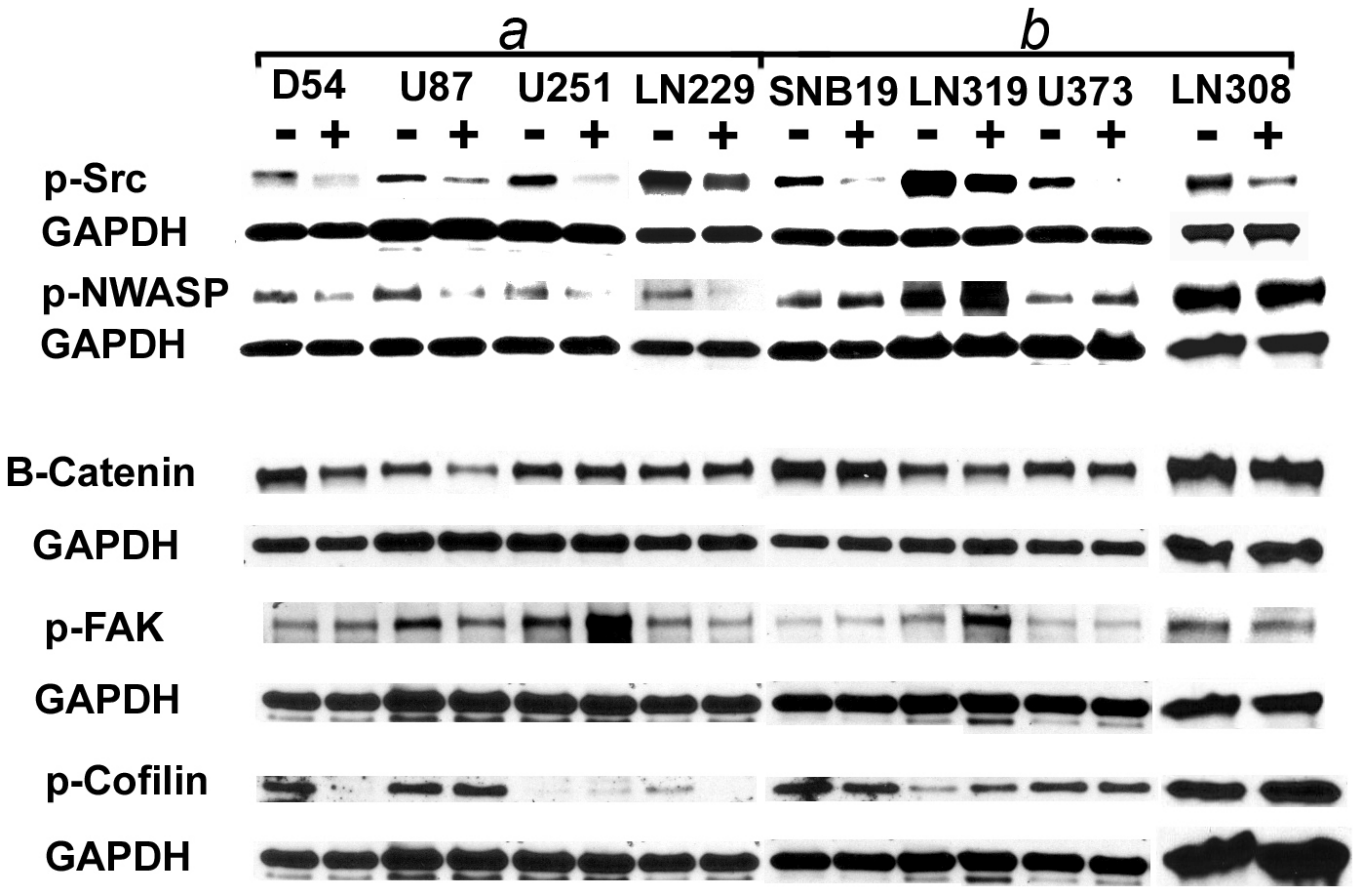
p-NWASP is preferentially-regulated by src in the cell lines that exhibit enhanced motility in low ambient oxygen conditions. Western analysis of p-src, p-NWASP, 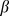-Catenin, p-FAK, and p-Cofilin after targeting src by siRNA in all 8 cell lines. 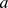 and *b* indicate the cell lines that do and do not exhibit the phenotype of increased motility under hypoxic conditions. (−) and (+) indicate negative control and src siRNA, respectively.

To determine which of p-NWASP, p-FAK-577, Cofilin and 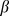-Catenin is more likely to partner with src in mediating the phenotype of increased motility under hypoxic conditions, we study their expression after targeting src in all eight cells lines. Interestingly, p-NWASP is the only molecule that is consistently downregulated in the cell lines that exhibit enhanced motility under hypoxic conditions but not in the cell lines that do not exhibit this phenotype. In particular, downregulating src lowers the levels of p-NWASP in U251, D54, LN229, and U87 cell lines, but not in SNB19, LN319, U373, and LN308 (see Figure 6). Notice also that none of the other 3 molecules separates the two groups of cell lines.

**Figure 7:**
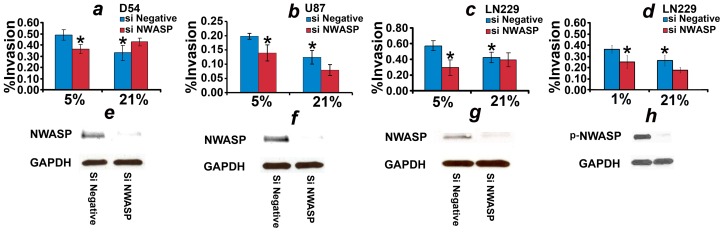
NWASP siRNA inhibits low oxygen-induced hypermotility. (a)-(c) plot, respectively, the matrigel motility data of D54, U87, and LN229, transfected by negative control (blue) and NWASP siRNAs (red) cultured in 5% and 21% ambient oxygen. (d) plots the matrigel motility data of LN229, transfected by negative control siRNA (blue) and the second NWASP siRNA (red) cultured in 1% and 21% ambient oxygen. (e)-(h) are western blots showing downregulation of NWASP/p-NWASP by siRNA in (a)-(d), respectively. * indicates a two-sample two-tailed ttest *P*<0.05 as compared to 5% or 1% negative control siRNA.

To study the effects of NWASP on the phenotype of low oxygen-mediated enhancement of motility, we target NWASP expression by siRNA and perform the matrigel motility assays. The data reveal that downregulating NWASP abrogates the enhancement of motility induced by 5% in D54, U87, and LN229 cell lines (see Figure 7). To obtain additional confirmation, we use a second NWASP siRNA and study LN229 cells in 1% ambient oxygen. The results also reveal that the second siRNA abrogates the enhancement of motility induced by 1% ambient oxygen (Figure 7). The NWASP siRNAs did not succeed in downregulating the expression of NWASP in U251 cells.

### 4.3 Motility in live brain sections

**Figure 8:**
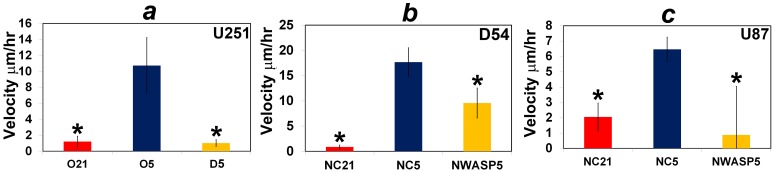
Dasatinib and NWASP siRNA abrogate the phenotype in live brain sections. The term “isolated” is used to describe a glioma cell that is not in contact with other tumor cells. (a) plots the mean velocity (*μ*m/hr) of isolated U251 cells that are the fastest moving in the plane from each of independent fields (20x magnification; a single cell is selected from each field); blue, red, and yellow indicate 5% oxygen (O5, 4 fields), 21% oxygen (O21, 4 fields), and 5% oxygen with 100 nM Dasatinib (D5, 5 fields), respectively. * indicates a two-sample two-tailed ttest *P*<0.05 as compared to O5. (b) and (c) plot the mean velocity of isolated D54 or U87 cells that are the fastest moving in the plane from each of independent fields (20x magnification; a single cell is selected from each field). In (b) and (c), blue, red, and yellow refer to, respectively, negative control (NC) siRNA under 21% oxygen (NC21; 3 fields for D54 or 4 fields for U87), NC siRNA under 5% oxygen (NC5; 8 fields for D54 or 5 fields for U87), and NWASP siRNA under 5% oxygen (NWASP5, 3 fields for D54 or 4 fields for U87). * indicates a two-sample two-tailed ttest *P*<0.05 as compared to NC siRNA in NC5. Illustrative time-lapse data are shown in Video S1.

Time-lapse microscopy of tumor cells in live brain sections is more realistic than *in vitro* motility assays because it recreates the complex architecture of the brain. Interestingly, the *in vivo* time-lapse data confirm the *in vitro* motility results. In particular, low oxygen increments the velocity of brain invasion by U251, D54 and U87 cells; furthermore, Dasatinib abrogates the phenotype in U251 cells while NWASP siRNA reduces the enhanced velocity of D54 and U87 cells in hypoxic conditions (see Figure 8 and supplementary time-lapse data, Video S1).

## Discussion

Here, we characterize a phenotype, observed in glioma cells, consisting of accelerated brain invasion under hypoxic conditions and identify Src and NWASP as mediators. Though, HIF1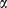 may play a role in general motility of gliomas (*ie* in normoxia) [32], [33], [34], [35], [36], [37], our findings support the idea that HIF1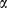 does not play a key role in enhancing motility in low ambient oxygen. In addition, the results reveal that src does not play a significant role in the general motility of glioma in the absence of low oxygen in 3/4 cell lines (*ie* U251, U87, and LN229; see Figure 1). In particular, D54 is the only cell line that shows a statistically-significant Dasatinib-mediated decrease in motility in 21% ambient oxygen (*p* = 0.0021).

The grow-or-go hypothesis assumes that cells have the ability to switch between the proliferative and invasive phenotypes depending on the oxygen concentrations in their local environment [38]; a cell may proliferate or invade but not both simultaneously. Tektonidis *et al.* applied lattice-gas cellular automata to model the phenotypic switch from proliferative to invasive; they report that the model, which best explains serial digital images of glioma spheroids implanted into collagen gel [39], assumes density-dependent phenotypic switching and repulsion between tumor cells [40]. Pham *et al.* modeled the density dependent phenotypic switch and found that it generates complex dynamics similar to those associated with tumor heterogeneity and invasion [41]. Hatzikirou *et al.* apply the lattice-gas cellular automata model to study the invasive phenotype of GBM; they propose that the grow-or-go phenotype and low oxygen conditions play a key role in the rapid growth of a GBM after resection [42]. Gerlee *et al.* propose a stochastic model of the grow-or-go phenotype such that the motile state is subject to random motion. They derive two coupled reaction-diffusion equations, which exhibit traveling wave solutions [43]. Here, we show that low ambient oxygen generates a src- and p-NWASP-mediated enhancement in motility/invasion of cultured cells in organotypic brain slice cultures and as measured by in vitro motility assays. Our results reveal that the threshold of oxygen that controls the phenotypic switch is higher than what is typically anticipated for cancer-related hypoxia; in fact, we observe enhancement in motility at 5% as well as 1% ambient oxygen. Because our experiments control ambient oxygen, we do not model the local switch between proliferative and invasive cells as a function of the local tumor cell densities. Technical limitations include the absence of a marker that distinguishes proliferative from invasive cells and the lack of accurate measures of the local oxygen concentrations in a tumor. The grow-or-go hypothesis predicts an inverse relationship between the extent of neovascularization and brain infiltration of GBM xenografts. Candolfi *et al.* found that, as compared to U251 tumors, U87 xenografts are less infiltrative and they exhibit profuse neovascularization without necrosis or hemorrhages [44]. These findings are consistent our results that low ambient oxygen enhances the invasion/motility of both U251 and U87 cells.

Actin filaments are organized in a Y-shaped branched array in lamellipodia; furthermore, actin-related protein 2/3 (Arp2/3) is localized at the branches [45]. WASP effects on actin are mediated by a carboxyl-terminal verpolin homology, Cofilin homology, acidic region (VCA) domain, which mediates WASP binding to the Arp2/3 complex. NWASP, cloned from a brain library, regulates actin polymerization by stimulating the actin-nucleation activity of the Arp2/3 [46], [47]. Active NWASP binds at the cleft between Arp2 and Arp3, thus holding them together in a closed form, to nucleate an actin filament [48], [49]. NWASP activation requires: 1) simultaneous binding of both Phosphatidylinositol 4,5-bisphosphate or PtdIns(4,5)P2 (PIP2) and Cell Division Control Protein 42 Homolog (cdc42), and 2) phosphorylation by Fak. NWASP contains a GTPase binding domain and a basic domain, which mediates binding to cdc42 and PIP2, respectively. Individually PIP2 and cdc42 are weak activators of NWASP; however, coincident signals from PIP2 and cdc42 unfold NWASP into the open state [50]. Interestingly, Keunen et al. also reported that low oxygen activates the phosphatidyl-inositol-3-kinase pathway in malignant glioma cells [7]. Open and unphosphorylated NWASP is translocated to the nucleus by interaction with importin. However, phosphorylation by Fak at Y256 causes decreased nuclear localization of WASP and improved cellular migration [51]. The open/phosphorylated WASP-Arp2/3 complex generates a dendritic array of filaments in lamellipodia by initiating new filaments or branches of F-actin at 70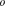 angles [52], [53], [45]. Cofilin is regulated by phosphorylation of a highly conserved serine residue; the dephosphorylated state is the active form [54]. Active Cofilin promotes debranching [55]. The data shown in Figure 4 are consistent with the idea that dephosphorylation of Cofilin promotes the phenotype by inhibiting debranching.

The aforementioned results are consistent with reports from other laboratories. Plasswilm et al. showed that low oxygen significantly increases motility of a GBM cell line (U1387-MG) in an *in vivo* chicken model [56]. Levin et al. studied 5 gliomas and reported that low oxygen elevates the levels of HIF1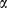, PARP1-cleaved Src, p-AKT-273, and p-AKT-308 [57]. In addition, NWASP has also been linked to breast cancer cell invasion [58], [59], [60].

Dasatinib does not cross the blood brain barrier; its accumulation in the brain is restricted by p-glycoprotein and breast cancer resistance protein and can be enhanced by Elacridar [61], [62]. Future experiments will determine the toxicity and optimal tolerated dose of Elacridar in brain-tumor bearing animals. The results will set the stage for animal survival experiments that test the hypothesis that Avastin and Dasatinib exert synergistic therapeutic effects. Finally, the findings are novel as they may be the first that characterize the molecular pathogenesis of the low oxygen-induced enhancement of motility by malignant gliomas.

## Supporting Information

Video S1 **Time-lapse microscopy of U251, U87, and D54 cells in organotypic brain slice cultures.** U251-O5, U251-O21 and U251-D5 indicate U251 cells cultured in 5% ambient oxygen, 21% ambient oxygen, and 5% ambient oxygen in the presence of Dasatinib, respectively. NC5, NC21, and NWASP5 indicate negative control siRNA and 5% ambient oxygen, negative control siRNA and 21% ambient oxygen, and N-WASP siRNA and 5% ambient oxygen, respectively. The experimental time is constant.
(AVI)
